# SARS-CoV-2 Infection Among Maternal-Infant Dyads in Ontario, Canada

**DOI:** 10.1001/jamanetworkopen.2021.20150

**Published:** 2021-08-09

**Authors:** Tiffany Fitzpatrick, Andrew S. Wilton, Hannah Chung, Astrid Guttmann

**Affiliations:** 1 Department of Epidemiology of Microbial Diseases, Yale School of Public Health, New Haven, Connecticut; 2 ICES, Toronto, Ontario, Canada

## Abstract

This cohort study uses population-based health data to assess SARS-CoV-2 testing outcomes among infants born in Ontario, Canada, during 9 months of the 2020 COVID-19 pandemic to mothers with confirmed infection at delivery.

## Introduction

Although reports of COVID-19 infection among infants are rare,^1,2,3^ many professional bodies have issued recommendations for the screening and management of neonates.^2,3,4^ Since April 1, 2020, polymerase chain reaction testing within 24 hours of birth has been recommended in Ontario—Canada’s largest province—for all infants born to mothers with confirmed SARS-CoV-2 infection at delivery.^2^ For this study, population-based birth registry, laboratory, and public health case data were assessed to describe SARS-CoV-2 testing outcomes among infants born during the COVID-19 pandemic and their mothers.

## Methods

We created a birth cohort of all infants delivered alive in Ontario during the COVID-19 pandemic period as identified either in the provincial hospital birth registry, MOMBABY, or SARS-CoV-2 laboratory data (for tested infants not yet registered in the birth registry). Mother-infant dyads were identified from MOMBABY records. The use of data in this project was authorized under section 45 of Ontario’s Personal Health Information Protection Act and exempt from research ethics board review. This study followed the Strengthening the Reporting of Observational Studies in Epidemiology (STROBE) reporting guideline.

We used linked administrative health, laboratory, and COVID-19 case management databases to identify SARS-CoV-2 diagnostic tests among all infants born in-hospital between February 1 and October 31, 2020, and maternal testing outcomes between January 15 and October 31, 2020.^5^ We summarized testing outcomes for maternal-infant dyads in the perinatal and postnatal period using SAS, version 9.4 (SAS Institute Inc). Specifically, we described the frequency of completed and positive tests reported among dyads and summarized the characteristics of infants tested for SARS-CoV-2. See the eMethods in the Supplement for additional details regarding these data sources.

## Results

In this cohort of 96 689 infants, 6176 (6.4%) had a record of receiving a diagnostic test for SARS-CoV-2; 1724 (1.8%) were tested perinatally (ie, within the first 2 weeks of life). Only 177 infants (0.1% of births; 2.9% of those tested) were positive for SARS-CoV-2 (Table). Median age at detection was 108 days (interquartile range, 50-189 days); fewer than 12 infections in infants (suppressed to maintain confidentiality) were identified perinatally. Of 177 infants infected with SARS-CoV-2, 90 (50.9%) had mothers who tested positive for SARS-CoV-2 at some point during the pandemic; however, only 6 (3.4%) were perinatal cases.

**Table.** Characteristics of Ontario Newborns Born Between February 1 and October 31, 2020, and Tested for SARS-CoV-2

Characteristic	No. (%)		
	Infants tested for SARS-CoV-2		Infants with laboratory-confirmed SARS-CoV-2 infection (n = 177)
	Any time since birth (n = 6176)	In the first 2 wk of life (n = 1724)	
SARS-CoV-2 tests, mean (SD), No. per child	1.25 (0.77)	1.29 (0.81)	1.29 (0.65)
Age at SARS-CoV-2 test, median (IQR), d	55.0 (10.0-143.5)	1.0 (0-5.0)	108.0 (50.0-189.0)
Sex			
Female	2693 (43.6)	761 (44.1)	71 (40.1)
Male	3421 (55.4)	928 (53.8)	99 (55.9)
Other or missing	62 (1.0)	35 (2.0)	7 (4.0)
Mother received SARS-CoV-2 test^a^			
2 wk Before birth	268 (4.3)	142 (8.2)	<6 (<3.4)^b^
On day of birth	717 (11.6)	601 (34.9)	12 (6.8)
2 wk After birth	363 (5.9)	279 (16.2)	<6 (<3.4)^b^
>2 wk After birth	1405 (22.8)	170 (9.9)	112 (63.3)
Not tested	2904 (47.0)	497 (28.8)	21 (11.9)
No maternal record identified	788 (12.8)	257 (14.9)	37-42 (20.9-23.7)^b^
Mother positive for SARS-CoV-2^a^			
2 wk Before birth	25 (0.4)	23 (1.3)	6 (3.4)^b^
On day of birth	22 (0.4)	22 (1.3)	
2 wk After birth	19 (0.3)	17 (1.0)	
>2 wk After birth	130 (2.1)	13 (0.8)	84 (47.5)
Time between mother’s and infant’s test, median (IQR), d^c^	0 (0-30)	0 (0-1)	0 (0-3)
Length of birth hospital stay, median (IQR), d	2 (1-3)	2 (1-10)	1 (1-2)

Abbreviation: IQR, interquartile range.

^a^ Categories are not mutually exclusive as some mothers received multiple tests.

^b^ Some cell sizes have been suppressed or data are reported in a range to reduce the risk of reidentification.

^c^ A positive value indicates that the infant was tested after the mother.

Only 156 of 82 484 delivering mothers (0.2%) were known to be positive for SARS-CoV-2 infection within 2 weeks of delivery. Only 6 infants (3.9%) born to these positive mothers were known to have acquired SARS-CoV-2 perinatally, and another 9 (5.8%) had positive tested results later in early infancy (Figure). Of note, 20 of 43 infants (46.5%) born to mothers known to be infected within 2 weeks of delivery had no record of being tested within 24 hours, as recommended by provincial guidelines.

**Figure.**
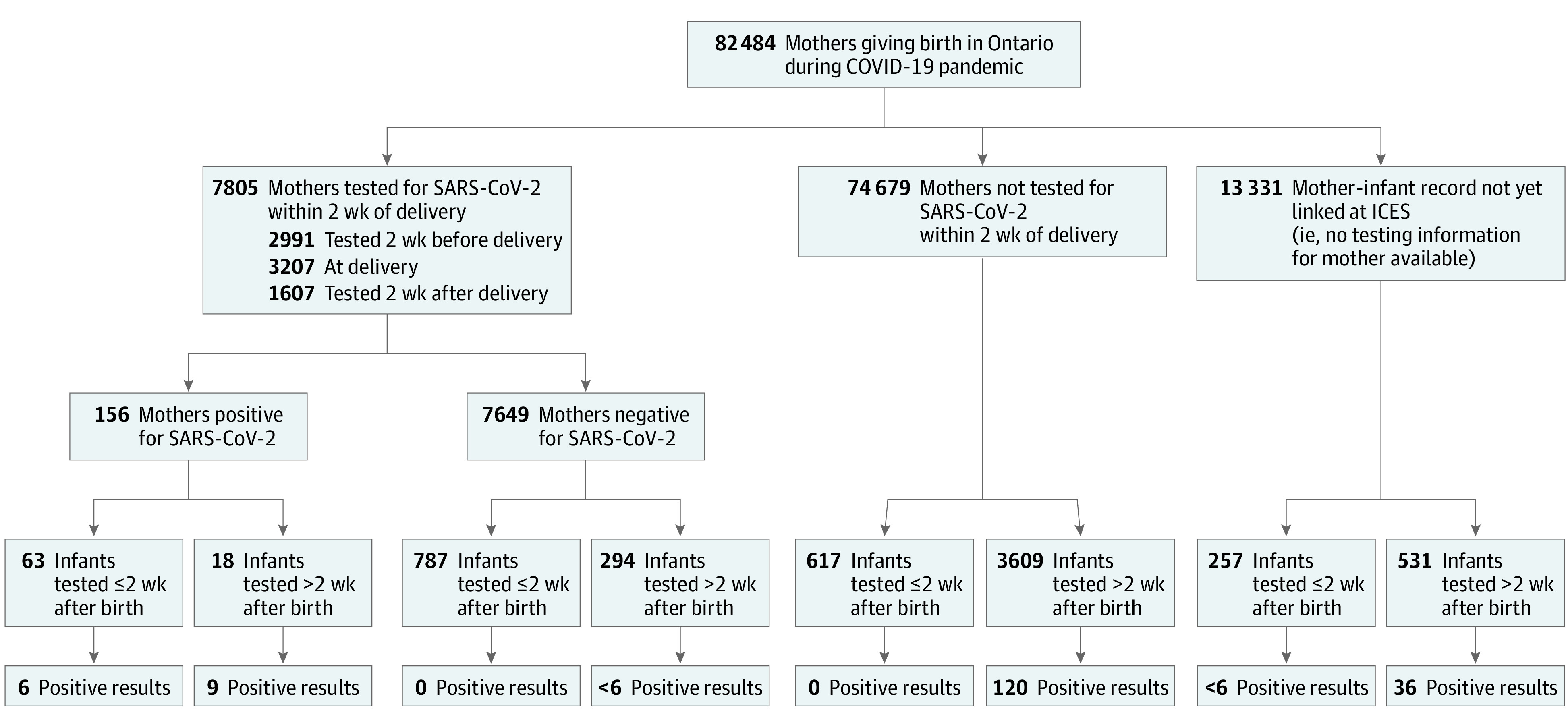
Flow Diagram for Perinatal SARS-CoV-2 Testing Among New Mother-Infant Pairs for Births Occurring in Ontario During the COVID-19 Pandemic Period (February 1 to October 31, 2020) Cell sizes representing fewer than 6 mothers or infants have been suppressed to reduce the risk of reidentification.

## Discussion

To our knowledge, this is the first population-based report of SARS-CoV-2 testing among a newborn cohort. The findings of this cohort study provide further evidence suggesting that perinatal transmission of, and early-life infection with, SARS-CoV-2 is rare. These findings align with an early report of a universal screening program for high-risk neonates and their parents^2^ and 2 recent studies from the US^1^ and Italy,^6^ none of which found evidence of vertical transmission.

Ontario’s current provincial guidelines do not recommend separation for newborns born to mothers confirmed to have SARS-CoV-2, although distancing and masks are recommended.^4^ These measures appear to have effectively limited transmission to newborns, without imposing potential harms through separation.^3^ It is not clear to what degree provincial testing guidelines were followed; 20 of 43 infants born to mothers known to be infected near delivery had no record of being tested within 24 hours. However, this evaluation is beyond the scope of our study.

Despite the strengths of its population-based nature, this study has limitations. Most notably, we were unable to link all maternal-newborn dyads, lacked data on stillbirths and pregnancy losses, and could not explicitly investigate vertical transmission. Furthermore, universal testing of new mothers and neonates is currently not recommended—consequently, leading to underestimation of the true incidence. In addition, we are unable to identify whether other household members had SARS-CoV-2, which likely is an important contributor to transmission once a mother-infant dyad is discharged. The findings of this cohort study suggest that SARS-CoV-2 infection in early infancy is rare; however, ongoing surveillance is required to ensure the continued protection of newborns from SARS-CoV-2 and its variants.

## Notes

Supplement. **eMethods.** Supplementary Methods
eReferences.
