# Supplementary material for: SARS-CoV-2 Infection Among Maternal-Infant Dyads in Ontario, Canada
Source: JAMA Netw Open. 2021 Aug 9;4(8):e2120150. doi: 10.1001/jamanetworkopen.2021.20150 (PMC8353536; doi:10.1001/jamanetworkopen.2021.20150)
Supplement: Supplement. — eMethods. Supplementary Methods eReferences. [file jamanetwopen-e2120150-s001.pdf]

Supplemental Online Content

Fitzpatrick T, Wilton AS, Chung H, Guttmann A. SARS-CoV-2 infection among maternal-infant dyads in Ontario, Canada. *JAMA Netw Open*. 2021;4(8):e2120150. doi:10.1001/jamanetworkopen.2021.20150

eMethods. Supplementary Methods

eReferences.

This supplemental material has been provided by the authors to give readers additional information about their work.

© 2021 Fitzpatrick T et al. *JAMA Network Open.*

eMethods. Supplementary Methods

Diagnostic tests for SARS-CoV-2 occurring during the period, defined as January 15 through October 31, 2020 (ie, latest date of data availability at the time of analysis), were identified from a combination of laboratory data sources, including the Ontario Laboratories Information System (OLIS), Distributed Labs (DL) data, Public Health Ontario (PHO) Labware data, and individuals confirmed positive from the integrated Public Health Information System/Case Contact Management System (iPHIS/CCM).

Notably, these data sets are updated and linked at different frequencies and, further, there may be delays in the reporting of outcomes from our data providers. The included laboratory databases are updated weekly, while MOMBABY was updated monthly and was approximately 99% complete through September 30, 2020, at the time of analysis. There are reporting delays from some hospitals; thus, not all relevant mother-infant pairs were yet linked, particularly for children born later in the study period for which reporting delays would be more likely. Similarly, some diagnostic tests occurring in Ontario during the study period might have not yet been included in these databases due to similar reporting delays. Notably, OLIS is a transactional database and reporting is not mandatory; thus, there is underreporting of the actual number of SARS-CoV-2 PCR tests performed in Ontario. Moreover, among the laboratories that contributed to OLIS, some did not contribute for the full study period. While an integrated data set was compiled to create a comprehensive COVID-19 testing data set, including OLIS and other sources of laboratory data, some diagnostic tests, such as those conducted by private diagnostic laboratories, are not included in these databases. Private testing is used for asymptomatic testing, such as in selected workplaces or for those needing documentation for travel, which is likely less relevant for the identification of tests among expectant mothers. As COVID-19 is a provincially mandated reportable disease, all individuals that meet the case definition are included in iPHIS; however, not all these individuals have been linked due to a lack of consent or necessary identifying information. Finally, testing data only capture individuals who have laboratory confirmed SARS-CoV-2. Thus, these results only represent a fraction of the actual number of SARS-CoV-2 infections. Although pregnant women have been a priority group for SARS-CoV-2 testing in Ontario since April 10, 2020,[1] symptomatic infections occurring after the perinatal period are likely undercounted.

These datasets included in this study were linked using unique encoded identifiers and analyzed at ICES, formerly the Institute for Clinical Evaluative Sciences.

eReference

1. Ontario Significantly expanding COVID-19 testing. News release. Ontario Newsroom. April 10, 2020. Accessed February 15, 2021. https://news.ontario.ca/en/release/56644/ontario-significantly-expanding-covid-19-testing

© 2021 Fitzpatrick T et al. *JAMA Network Open.*
